# Genetic Heterogeneity Underlying Familial Short Stature

**DOI:** 10.3390/diagnostics15243127

**Published:** 2025-12-09

**Authors:** Margot Comel, Mouna Barat-Houari, Fanny Alkar, Cyril Amouroux, Olivier Prodhomme, Nathalie Ruiz, Sophie Rondeau, Constance F. Wells, Yves-Marie Pers, David Geneviève, Marjolaine Willems

**Affiliations:** 1 Montpellier University, ERN ITHACA, Génétique Clinique, Département de Génétique Médicale, Maladies Rares et Médecine Personnalisée, Centre Hospitalier Universitaire de Montpellier, Centre de Référence Constitutif des Maladies Osseuses Constitutionnelles, 34295 Montpellier, France; margot.comel@chu-montpellier.fr (M.C.);; 2 Department of Molecular Genetics and Cytogenomics, University Hospital of Montpellier, 34295 Montpellier, France; 3 Unité d’Endocrinologie et Gynécologie Pédiatriques, Service de Pédiatrie, Hôpital Arnaud de Villeneuve, CHU de Montpellier et Université Montpellier, 34295 Montpellier, France; 4 Département d’Imagerie Pédiatrique, Centre Hospitalier Universitaire de Montpellier, 34295 Montpellier, France; 5 Université de Paris, Institut IMAGINE, Molecular and Physiopathological Bases of Osteochondrodysplasia, INSERM UMR1163, 75015 Paris, France; 6 Genomic Medicine Service for Rare Disease, Necker-Enfants Malades Hospital, AP-HP, 75015 Paris, France; 7 Institute for Regenerative Medicine and Biotherapy, University of Montpellier, INSERM UMR 1183, 34095 Montpellier, France; 8 Clinical Immunology and Osteoarticular Diseases Therapeutic Unit, University Hospital of Montpellier, 34295 Montpellier, France

**Keywords:** short stature, dyschondrosteosis, acrodysostosis, acroscyphodysplasia, *SHOX*, *PDE4D*, ACAN

## Abstract

**Background and Clinical Significance:** Familial short stature is a common reason for referral in clinical genetics. While often attributed to a single genetic cause, genetic heterogeneity can complicate diagnosis and management. This report describes a family in which three distinct pathogenic variants in *SHOX*, *PDE4D* and *ACAN* caused overlapping phenotypes of familial short stature. **Case Presentation:** Clinical, radiological and molecular data were collected retrospectively at the Reference Centre for Constitutional Bone Diseases at Montpellier University Hospital. Targeted gene panels, whole genome sequencing and Sanger sequencing were employed to identify pathogenic variants. Variant interpretation followed the guidelines of the American College of Medical Genetics. A pathogenic *SHOX* variant (c.452G>A; p.Ser151Asn) was identified in the proband and her mother, which is consistent with dyschondrosteosis. A de novo *PDE4D* variant (c.671C>T; p.Thr224Ile) was identified in a cousin presenting with syndromic acrodysostosis. An *ACAN* splice variant (c.6833-1G>A) was detected in several family members and is associated with short stature and skeletal anomalies. An individual carrying both the *SHOX* and *ACAN* variants exhibited a more severe phenotype, suggesting an additive effect. **Conclusions:** This case study highlights the importance of systematic molecular investigations in families with overlapping yet heterogeneous phenotypes. Comprehensive genetic familial analysis enables personalized care and accurate genetic counselling, particularly when multiple diagnoses coexist. A family history should not preclude molecular testing, since similar phenotypes can result from different genetic causes.

## 1. Introduction

Short stature is defined as a height more than two standard deviations (SD) below the population mean for age and sex. Growth is a complex process influenced by genetics, nutrition, hormones and environmental factors. Genetic variation plays a major role, accounting for roughly 60–80% of inter-individual differences in linear growth. More than 700 genes have been involved in its regulation [1].

A significant proportion of children diagnosed with idiopathic short stature have genetic factors contributing to their condition. Advances in sequencing technologies have increased both the diagnostic yield and the speed of genetic testing [2].

Skeletal dysplasia, also known as osteochondrodysplasia, encompasses a spectrum of skeletal disorders resulting from abnormal bone development and growth [3]. The 11th updated nosology of genetic skeletal disorders by the Nosology Group of the International Skeletal Dysplasia Society (ISDS) has identified and categorized up to 771 distinct entities into 41 groups [4]. The global incidence is estimated at 1 in 5000 births [3]. By 2023, the number of identified genes had reached 552 in the revised nosology. These genes exhibit functional diversity, influencing a wide array of biological processes and being affected by various mutational mechanisms [4].

Nevertheless, the diagnostic odyssey may not end with the identification of a single genetic cause. In individuals who carry two pathogenic variants (PVs) in distinct genes, the clinical phenotype may represent an overlap of the features associated with each condition [5].

In this article, we retrospectively described a family with short stature caused by three different PVs in Short stature homeobox (*SHOX*, OMIM 32865), Phosphodiesterase 4D (*PDE4D*, OMIM 600129) and Aggrecan (*ACAN*, OMIM 155760).

## 2. Materials and Methods

Clinical report: We collected clinical, molecular and immunological data from individuals at the reference center for Constitutional Bone Disease (CBD) in Montpellier University Hospital, France. All patients were evaluated and followed at Montpellier University Hospital. Clinical data were collected from medical record review and interviews with individuals, parents and physicians. All individuals gave their written informed consent before molecular analysis and subsequently to be included in this retrospective study. The study adhered to the Declaration of Helsinki protocol. Patients are reported in this study in the chronological order in which they were seen in the Genetics department.

Molecular Analysis: Standard procedures were used to isolate DNA from peripheral blood samples of all affected members and their relatives. Genomic DNA was extracted from 1 mL of whole blood using the QIAsymphony Midi Kit (Qiagen, Hilden, Germany), following the manufacturer’s protocol. Samples were eluted in a final volume of 200 µL of AE buffer.

Targeted molecular study of *SHOX* was conducted by analyzing the PAR1 region, including *SHOX*, using Multiplex Ligation-dependent Probe Amplification SALSA MLPA Kit P018-F1 *SHOX* (MRC Holland, Amsterdam, The Netherlands) at Cerba laboratory. The coding regions (exons 2 to 6a) were analyzed by direct sequencing of PCR products after gene amplification.

Comprehensive molecular analyses were performed using targeted gene panels at two centers. Individual V.1 was analyzed using version 5 of the CBD subpanel of the osteochondrodysplasias panel (162 genes) at Necker Hospital, Paris. Individuals IV.5, IV.7, IV.3, III.2, III.5 and III.9 had the CBD panel (82 genes; https://umai-montpellier.fr/doc/Panel_MOC [accessed on 27 October 2025]) at the molecular biology laboratory in Montpellier University Hospital, or by targeted Sanger sequencing in the same laboratory.

Library preparation was performed using the Twist Library Preparation Kit (Twist Bioscience, South San Francisco, CA, USA) (Montpellier) or standard Illumina protocols (Necker), followed by paired-end sequencing on NextSeq500 (Illumina, San Diego, CA, USA). Target region coverage was evaluated to ensure that at least 95% of regions had an average read depth of 100x or higher. FASTQ files were aligned to the human reference genome (GRCh37/hg19), provided by the University of California Santa Cruz (UCSC) database.

Variant calling was performed using GATK HaplotypeCaller (broadinstitute.org) and Google DeepVariant (Montpellier, France) or SAMtools [6] and GATK [7] (Necker). Copy number variant (CNV) analysis was performed using GATK4 GermlineCNVCaller module with depth coverage normalization. Variants were annotated and filtered using in-house software systems (Polyweb at Necker, unpublished; MobiDL pipeline at Montpellier, https://github.com/mobidic/MobiDL [accessed on 1 March 2024]). Analyses focused on non-synonymous variants, splice variants, and coding indels.

Variant pathogenicity was evaluated using SIFT, PolyPhen-2, and CADD prediction algorithms. Variant frequencies were assessed using gnomAD and in-house databases. Filtering criteria included: (i) dominant mode of inheritance, (ii) genotype/phenotype correlation, and (iii) minor allele frequency (MAF) threshold of 0.1%. Variants were classified according to the American College of Medical Genetics (ACMG) guidelines [8] using Mobidetails, Franklin by Genoox and VarSome.

All variants were confirmed by Sanger sequencing (Applied Biosystem v3.1). Familial segregation analysis was performed whenever possible to evaluate inheritance patterns and de novo occurrence.

## 3. Results

**Figure 1:**
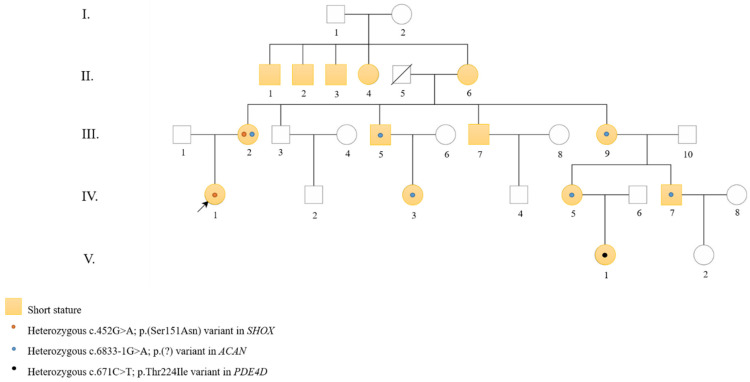
Pedigree of the family. Squares indicate males and circles indicate females. Filled yellow symbols represent individuals with short stature. An arrow indicates the proband. Orange dots denote individuals carrying the *SHOX* variant, blue dots indicate individuals carrying the *ACAN* variant and black dot marks the only *PDE4D* carrier.

Clinical report (Figure 1, Table 1 and Table 2):

The family pedigree is shown in Figure 1 and illustrates the segregation of the different PVs within the family. Table 1 summarizes the clinical features of family members with short stature and carrying PVs in one of the three genes: *SHOX*, *ACAN*, and *PDE4D*.

**Figure 2:**
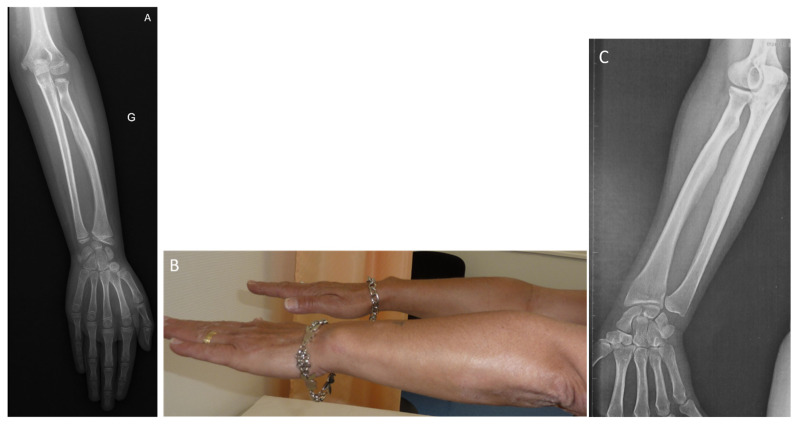
(**A**) Radiograph of the left forearm of individual IV.1 at 11 years and 7 months showing medial inclination of the distal radial epiphysis (Madelung deformity) as well as a triangularization of the carpus resulting from the inclination of the radial epiphysis. G is “left” in french (gauche). (**B**) Photograph of the forearms of individual III.2 (mother of individual IV.1) at 43 years old showing medial inclination of the distal radial epiphysis (Madelung deformity). (**C**) Radiograph of the right forearm of individual III.2 (mother of individual IV.1) at 43 years old showing medial inclination of the distal radial epiphysis (Madelung deformity).

The proband (individual IV.1) was diagnosed at the age of 11 due to short stature and was referred to the medical genetics department in Montpellier. The individual’s parents were unrelated. The mother (individual III.2) was 140 cm in height (−4.0 SD), while the father (individual III.1) was 180 cm (+0.8 SD). Parental target height was 153.5 cm (−1.7 SD). At 39 weeks’ gestation, her birth length was 46 cm (−1.7 SD), her birth weight 2680 g (−1.4 SD), and her head circumference 35 cm (+0.6 SD). At 11 years of age, her height was 129 cm (−2.7 SD), her weight 30 kg (−1.0 SD) and her head circumference 53 cm (0 SD, mean). Her wingspan to height ratio was 0.94. Clinically, she had astigmatism and myopia. She exhibited brachymesophalangia affecting both hands and radiographic evidence of dyschondrosteosis, as shown in Figure 2A. There was a discrete mesomelic shortening.

Individual III.2 was 140 cm tall (−4.0 SD). She had mesomelic shortening, scoliosis and genu varum. She had bilateral brachymesophalangia of the fifth finger and a discrete Madelung deformity as seen in Figure 2B. Additionally, she has been diagnosed with psoriasis and hyperthyroidism. She also had a late puberty. X-rays taken at the time of her daughter’s diagnosis demonstrated consistent findings with dyschondrosteosis as depicted in Figure 2C.

A cousin of the proband (individual IV.5), unrelated to her partner, had a pregnancy marked by intrauterine growth retardation (IUGR). The first trimester ultrasound revealed a nuchal translucency of 2 mm and a cephalo-caudal length of 56.5 mm. The third trimester ultrasound revealed a delayed bone growth, with a femoral and humeral length below the third percentile. Individual IV.5 had genetic counseling and was reassured during pregnancy by the history of short stature in the family, noted in several relatives, including herself. No invasive examination, such an amniocentesis, was proposed.

**Figure 3:**
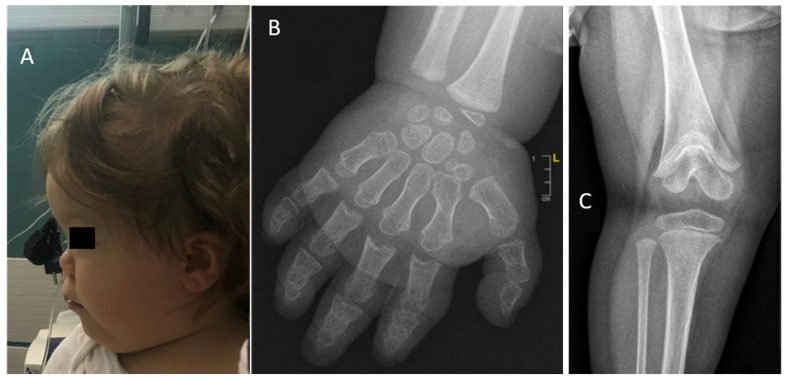
(**A**) Profile photograph of individual V.I at 2 years old showing features similar to photograph (**A**) as well as low-set ears. (**B**) Radiograph of the right hand of individual V.1 at 18 months showing acrodysostosis, brachymetacarpy and brachydactyly, cone-shaped epiphyses. Bone age is also very advanced (about 5 years in this radiograph). (**C**) Radiograph of the right knee of individual V.1 at 18 months showing cup-shaped metaphyses with embedded epiphyses (specific radiological appearance of acroscyphodysplasia).

A female newborn (individual V.1) was delivered at 38 weeks’ gestation, with a height of 43 cm (−2.8 SD), a weight of 2130 g (−2.3 SD) and a head circumference of 31 cm (−2.0 SD). The mother (individual IV.5) was 150 cm (−2.3 SD) and the father (individual IV.6) was 170 cm (−0.8 SD), her target height of 153.5 cm (−1.7 SD). This small child grew up with development delays, unlike the other family members. Clinical features are shown in Figure 3A. A clinical and radiological examination revealed acrodysostosis with abnormalities of the extremities, particularly the hands, stiffness of the knees with a typical aspect of acroscyphodysplasia on the right knee and significant bone age advance. Radiological findings are depicted in Figure 3B,C. At the age of two, her height was 81 cm (−1.9 SD), her weight 11.5 kg (0 SD, mean), and her head circumference 47.5 cm (0 SD, mean). The wingspan to height ratio was 0.9. In addition, she was affected by asthma, obstructive sleep apnea, divergent strabismus, Chiari anomaly and obesity. She was prone to recurrent infections of the ear, nose, and throat (ENT). At the age of 18 months, she was diagnosed with osteomyelitis, a bone infection that required hospital treatment.

The brother of patient IV.5, individual IV.7, measured 162 cm (−2.2 SD), weighted 57 kg (−1.1 SD) and had a head circumference of 53.5 cm (−2.5 SD). The wingspan to height ratio was 1.02. The individual had a high palate, an L5-S1 disc anomaly, left clavicular subluxation and patellar malformation. His birth height was 41 cm at 36 weeks’ gestation (−2.9 SD).

The mother (individual III.9) of individuals IV.5 and IV.7 was 144 cm tall (−3.4 SD), weighted 53 kg (0 SD, mean) and had a head circumference of 52.5 cm (−2.0 SD). The wingspan to height ratio was 1.04. The individual had osteoarthritis, joint mobility in the knees with a notion of deformity in the left knee, as well as ligament problems.

**Figure 4:**
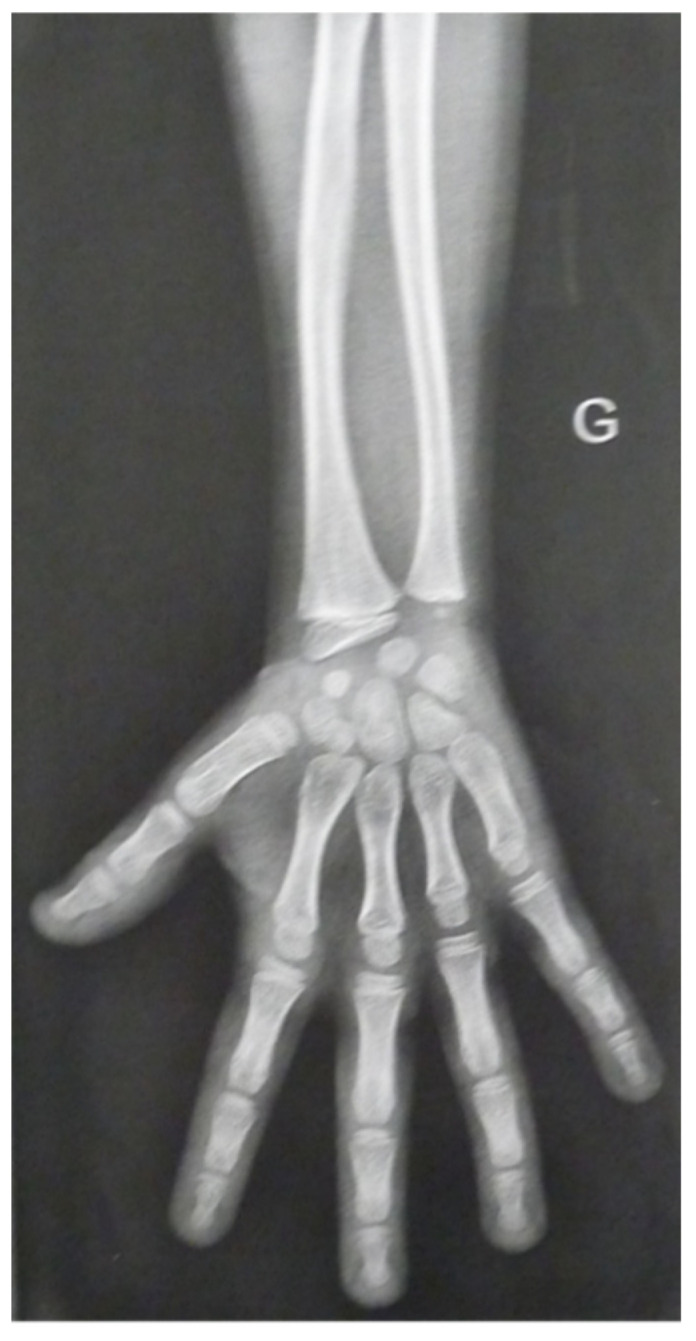
Radiograph at 7 years and 5 months of the left forearm of individual IV.3 showing slight brachymetacarpy of the 4th and 5th fingers. G is for “gauche”, it is “left” in french.

A further maternal cousin of the proband (individual IV.3) was also referred because of her short stature when she was 7. Her parents were not related. Her mother (individual III.6) was 153 cm in height (−1.8 SD) and her father (individual III.5) was 165 cm in height (−1.6 SD). The parental target height was therefore 152.5 cm (−1.8 SD). She already had IUGR since at birth, she was 43 cm in length (−3.3 SD), weighed 2185 g (−2.6 SD), and had a head circumference of 31 cm (−2.4 SD). At the age of 11, her height was measured at 122.7 cm (−3.4). She exhibited a body weight of 24.5 kg (−1.8 SD), with a head circumference of 50 cm (−2.5 SD). The wingspan to height ratio was 1.05. She had learning difficulties at school, attention deficit disorder and a discrete brachymetacarpy as illustrated in Figure 4. At 12, a psychometric assessment yielded a total intelligence quotient between 61 and 73. At the age of 13, she was invited by the medical genetics team to participate in the French genomic medicine pilot project DEFIDIAG, which involved trio genome sequencing [9].

Molecular analysis: A *SHOX* gene sequencing was carried out in individual IV.1 and a variant was identified: c.452 G>A; p.Ser151Asn (NM_000451). It is classified as pathogenic according to ACMG criteria [8]. This variant was absent from control population databases (GnomAD, dbSNP). According to MetaDome, this variant’s position is highly intolerant to variation (MetaDome Version 1.0.1). Various prediction software programs favor a damaging variant (PolyPhen 2 [10], SIFT [11], MISTIC [12], ClinPred [13], Meta LR [14]). This variant was also identified in the individual’s mother (individual III.2).

Given the syndromic association in individual V.1, a molecular analysis of the genes involved in acrodysostosis was carried on a CBD panel. A de novo variant was subsequently identified in *PDE4D,* c.671 C>T; p.Thr224Ile (NM_001104631), explaining the child’s syndromic phenotype (Figure 3A,B). It is classified as likely pathogenic according to ACMG criteria [8]. This variant was absent from the control population databases (GnomAD, dbSNP). According to MetaDome, this variant’s position is highly intolerant to variation (MetaDome Version 1.0.1). Prediction software programs are in favor of a damaging variant (SIFT [11], MISTIC [12], ClinPred [13], Meta LR [14], REVEL [15]). No other variant, in particular in *SHOX* and *ACAN*, was identified.

In her mother, individual IV.5, targeted testing for familial PVs in *PDE4D* and *SHOX* were negative.

A CBD panel was carried out on individual IV.7, brother of individual IV.1 and their mother (individual III.9), and an *ACAN* variant, c.6833-1 G>A; p.? (NM_013227), was identified in both cases. It is classified as likely pathogenic according to ACMG criteria [8]. This variant was absent from control population databases (GnomAD, dbSNP). This is a null variant and loss of function is a known mechanism of pathogenicity [16,17]. No *SHOX* variant was identified in both individuals. A targeted test revealed the same *ACAN* genetic variation in individual IV.5. It was also subsequently verified that the child V.1 did not carry the pathogenic *ACAN* variation identified in her mother.

The proband’s *SHOX* variant was first not found in patient IV.3. Family investigations revealed that individual III.5 was also a carrier of the *ACAN* variant. His daughter, individual IV.3, also carried this variant. Subsequently, a whole genome sequencing was performed to investigate the cause of her intellectual disability, but failed to identify any pathogenic or probably pathogenic variants responsible for her neurodevelopmental disorder. Thereafter, it was discovered that individual III.2 carried the familial variant in *ACAN*. She therefore carried two PVs that may explain her short stature.

## 4. Discussion

The short stature observed in this family is due to variants in three different genes, illustrating the critical importance of obtaining molecular evidence when presented with similar phenotypes within a family, even when a genetic variant has already been identified in other members. The proband’s clinical presentation is attributable to a PV in *SHOX*, typically associated with short stature and Madelung deformity, with no extra-osseous elements [18]. However, comprehensive molecular analysis revealed that not all family members with short stature shared this genetic etiology. A *PDE4D* variant was identified using a CBD panel in a cousin of the proband, who exhibited both short stature and developmental delay. This variant explained her syndromic phenotype, which include specific morphological features, endocrine disorders and variable intellectual disability, among others [19], differing fundamentally from the isolated skeletal manifestations seen in *SHOX* deficiency. A similar CBD panel conducted on the subject’s mother, uncle, and maternal grandmother revealed an *ACAN* variant not previously identified in the family. Furthermore, a second cousin of the proband, who exhibited similar characteristics but did not carry the *SHOX* variant, was identified as having the same *ACAN* variant. PV in *ACAN* may be associated with early osteoarthritis (adolescence, early adulthood) and early disc degeneration [20], representing yet another distinct clinical trajectory that requires specific long-term monitoring. These three diagnoses imply very different clinical management and genetic counselling strategies. Apart from the growth phenotype, the extra-osseous symptoms differ significantly, necessitating tailored surveillance and therapeutic approaches for each genetic subgroup within the family. It is therefore important to provide the biology laboratory performing the molecular analysis with detailed phenotypes, as insufficient clinical information can make variant interpretation difficult and may lead to incomplete diagnostic workup.

This study report three novel variants. The *SHOX* variant is a missense variant in a Homeobox DNA-binding domain [21]. *SHOX* is located in the pseudoautosomal region (PAR), which explains the short stature seen in Turner syndrome. Expression of the gene appeared to be restricted to osteogenic cells [22]. *SHOX* encodes a homeodomain, which serves as the DNA-binding motif for eukaryotic transcription factors, facilitating additional crucial functions like nuclear localization and protein–protein interactions. The majority of genes containing homeobox sequences participate in developmental regulation, differentiation, and organogenesis, presenting varied expression patterns across spatial and temporal contexts [23]. Heterozygous *SHOX* PVs are responsible for autosomal dominant conditions including Leri-Weill dyschondrosteosis (LWD, OMIM 127300). To address short stature, growth hormone therapy can be initiated in prepubertal children [24]. Madelung’s deformity, if painful, can be treated conservatively with the use of splints or, in more severe cases, surgically [25]. This condition is not considered severe or eligible for prenatal and/or pre-implantation diagnostic procedures under French law. The clinical manifestations observed in carrier family members reported here are consistent with the classical phenotype of this condition.

The *PDE4D* variant is a missense variant and is not in a defined protein domain. PDE4D is a member of the cyclic AMP (cAMP) hydrolyzing phosphodiesterase family, which directly influences the rate of cAMP degradation. Given the pivotal role of cAMP in intracellular signaling triggered by various membrane-impermeable hormones, it can be hypothesized that dysregulation of cAMP levels may underlie the acrodysostosis associated with *PDE4D* variations [19]. Pathogenic heterozygous variations in this gene are responsible for acrodysostosis 2, with or without hormone resistance (ACRDYS2, OMIM 614613) [26] also known as inactivating PTH/PTHrP Signaling Disorder type 5 (iPPSD-5) according to the new classification [27]. Acrodysostosis involves skeletal characteristics, including short stature, facial dysostosis with nasal hypoplasia, and peripheral dysostosis, featuring severe brachymetatarsia, brachymetacarpy, brachydactyly, cone-shaped epiphyses, and advanced bone maturation. Additionally, there may be inconsistent resistance to various hormones, such as parathyroid hormone or thyrotropin, which is rare, and a frequent neurological involvement leading to mild to moderate intellectual disability [28]. *PDE4D*-related acrodysostosis includes acroscyphodysplasia, characterized by a typical knee appearance and a more severe prognosis [4,26]. Furthermore, numerous case–control studies have demonstrated a correlation between *PDE4D* variants and the risk of ischemic stroke across diverse ethnic groups [29]. Individuals with a *PDE4D* variant require comprehensive, multidisciplinary long-term follow-up, including assessment of cardiovascular and thromboembolic risk factors [26]. In contrast to LWD, this condition is regarded as incurable and significantly severe in France. Consequently, this condition qualifies for both prenatal and pre-implantation genetic diagnosis.

The *ACAN* variant affects gene splicing (dbscSNV [30]). Aggrecan is a major component of cartilage extracellular matrix [31]. This proteoglycan is found in growth plate cartilage, articular cartilage and intervertebral disc cartilage [32]. A heterozygous PV in this gene leads to autosomal dominant diseases such as spondyloepiphyseal dysplasia Kimberley type (SEDK, OMIM 608361). The clinical presentation includes short stature, a robust physique, and the early onset of osteoarthritis in weight-bearing joints. Radiographically, characteristic features include flattened vertebral bodies with sclerosis and irregular end plates, along with flattened femoral epiphyses [33]. Another syndromic presentation associated with *ACAN* is short stature and advanced bone age, with or without early-onset osteoarthritis and/or osteochondritis dissecans (SSOAOD, OMIM 165800). The clinical features of this disease include osteochondritis dissecans, premature osteoarthritis and mild, disproportionate short stature [34]. Additionally, there may be facial morphological features, including prognathia, mid-facial hypoplasia, posteriorly rotated ears, and relative macrocephaly. Brachydactyly, genu valgum, and other joint problems may also be present [35,36]. The clinical presentation of the family is in favor of this diagnosis. Growth therapies, such as growth hormone treatment, could be beneficial in improving the adult height of affected individuals [37,38,39,40].

During the antenatal period, ultrasound scans are unable to visualize the full phenotype and may fail to identify significant syndromic elements. A diagnosis can then be evoked without molecular analysis if one of the parents is a carrier of a similar phenotype. However, as demonstrated in this family, clinical similarity does not guarantee genetic identity, raising significant ethical considerations regarding prenatal testing strategies. The genetic conditions identified in this family have different implications for prenatal and preimplantation diagnosis (PGD) under French law [41]. According to French bioethical legislation, PGD and medical termination of pregnancy (MTP) are authorized exclusively for particularly severe conditions that are deemed incurable at the time of diagnosis. In this family, the parents had the option undergoing a MTP if the presence of the *PDE4D* variant had been detected antenatally. Acrodysostosis type 2 is considered of particular gravity due to its syndromic nature, including variable intellectual disability, and multiple organ involvement. Conversely, heterozygous *SHOX* or *ACAN* variants are not considered of particular gravity under French law. The condition does not affect life expectancy, cognitive function, or overall quality of life, in a manner that would justify MTP. This regulatory framework raises important ethical questions regarding the boundaries between “treatable” and “incurable” conditions, and the definition of severity. The concept of “particular gravity” remains open to interpretation and may evolve with medical advances. The case of this family underscores the critical importance of accurate molecular diagnosis before any prenatal counseling.

An additive effect of the *SHOX* and *ACAN* PVs may be suspected, given the short stature (−4.0 SD) severity in individual III.2 carrying both PVs, compared with other family members carrying a *SHOX* variant alone (−2.7 SD) or an *ACAN* variant alone (size from −1.6 to −3.4 SD). The phenotype of a patient with dual genetic diagnoses may be influenced by the extent to which the phenotypes associated with each individual condition overlap [5]. Both *SHOX* and *ACAN* are involved in bone development and growth, acting through distinct yet potentially interconnected mechanisms that converge on the regulation of skeletal growth, particularly through cartilage biology and chondrocyte differentiation. Aggrecan, a key constituent of the cartilage extracellular matrix, is regulated through the synergistic action of SHOX and the SOX transcription factors (SOX5, SOX6, and SOX9), with protein–protein interactions between SHOX and SOX5/SOX6 playing a central role [42,43,44]. Thus, a decrease in *SHOX* expression may result in a decrease in *ACAN* expression, potentially leading to a more severe phenotype than a loss of *ACAN* monoallelic function mutation alone. However, direct evidence of synergistic effects in humans remains limited. These hypotheses therefore remain assumptions.

## 5. Conclusions

This study highlights the importance of systematic and unbiased molecular analysis of several individuals from the same family when phenotypes are similar but not identical, in order to provide the best personalized patient management and genetic counselling. The presence of a family history should not lead to a diagnosis without suggesting molecular analysis.

## Figures and Tables

**Table 1:** Summary of the clinical and genetic characteristics of individuals of short stature and carrying a pathogenic variant in the family.

Individual	Clinical Features	Height (SD)	Identified Variant(s)	Gene(s) Involved	Zygosity/Inheritance
IV.1 (proband)	Short stature, brachymesophalangia, dyschondrosteosis, myopia, astigmatism	−2.7 SD at age 11	c.452G>A; p.Ser151Asn	*SHOX*	Heterozygous, maternal
III.2	Short stature, mesomelia, scoliosis, genu varum, brachymesophalangia, dyschondrosteosis, Madelung deformity, psoriasis, hyperthyroidism, late puberty	−4.0 SD	c.452G>A; p.Ser151Asn c.6833-1G>A; p.?	*SHOX*, *ACAN*	Heterozygous for both
IV.5	Short stature	−2.3 SD	c.6833-1G>A; p.?	*ACAN*	Heterozygous, maternal
V.1	Short stature, developmental delay, acrodysostosis, cone-shaped epiphyses, acroscyphodysplasia, bone age advancement, asthma, OSA, Chiari anomaly, ENT infections, obesity, divergent strabismus, pyelonephritis, osteomyelitis	−1.9 SD at age 2	c.671C>T; p.Thr224Ile	*PDE4D*	Heterozygous, de novo
IV.7	Short stature, spinal/clavicle/patella anomalies, high palate	−2.2 SD	c.6833-1G>A; p.?	*ACAN*	Heterozygous, maternal
III.9	Short stature, osteoarthritis, hypermobility, knee deformity, ligament issues	−3.4 SD	c.6833-1G>A; p.?	*ACAN*	Heterozygous
IV.3	Short stature, ADHD, learning difficulties, IQ 61–73	−3.4 SD at age 11	c.6833-1G>A; p.?	*ACAN*	Heterozygous, paternal
III.5	Not clinically described	−1.6 SD	c.6833-1G>A; p.?	*ACAN*	Heterozygous

SD: standard deviation; OSA: obstructive sleep apnea; ENT: ear, nose and throat; ADHD: attention-deficit/hyperactivity disorder; IQ: intelligence quotient.

**Table 2:** Summary of the main phenotypic features associated with each variant in the family. Data are derived from clinical evaluations of affected family members with molecular diagnosis. An additive effect is suspected in individual III.2.

Mutated Genes	*SHOX* c.452G>A; p.Ser151Asn	*SHOX* and *ACAN*	*ACAN* c.6833-1G>A; p.?	*PDE4D* c.671C>T; p.Thr224Ile
Number of carriers	1 (IV.1)	1 (III.2)	5 (IV.3, IV.5, IV.7, III.5 and III.9)	1 (V.1)
Mean height	−2.7 SD	−4.0 SD	−2.6 SD (−3.4 to −1.6)	−1.9 SD
Main skeletal features	Mesomelic shortening, brachymesophalangia, dyschondrosteosis	Mesomelic shortening, scoliosis; genu varum, brachymesophalangia, dyschondrosteosis, Madelung deformity	Mild rhizo-mesomelic shortening, joint anomalies, early-onset osteoarthritis	Acrodysostosis, cone-shaped epiphyses, acroscyphodysplasia, bone age advancement
Other clinical findings	Myopia, astigmatism	Psoriasis, hyperthyroidism, late puberty	High palate, hypermobility, ligament issues	Developmental delay, Chiari malformation, ENT infections, asthma, OSA, obesity, strabismus, pyelonephritis, osteomyelitis

SD: standard deviation; ENT: ear, nose and throat; OSA: obstructive sleep apnea.

## Data Availability

The raw data supporting the conclusions of this article will be made available by the authors on request.
